# The impact of maternal morbidity on cesarean section rates: exploring a Latin American network of sentinel facilities using the Robson’s Ten Group Classification System

**DOI:** 10.1186/s12884-023-05937-3

**Published:** 2023-08-24

**Authors:** Claudio Sosa, Bremen de Mucio, Mercedes Colomar, Luis Mainero, Maria L. Costa, Jose P. Guida, Renato T. Souza, Adriana G. Luz, José G. Cecatti, Maria H. Sousa, Carmen M. Cruz, Luz M. Chevez, Rita Lopez, Gema Carrillo, Ulises Rizo, Erika E. Saint Hillaire, William E. Arriaga, Rosa M. Guadalupe, Carlos Ochoa, Freddy Gonzalez, Rigoberto Castro, Allan Stefan, Amanda Moreno, Suzanne J. Serruya

**Affiliations:** 1 Latin American Center of Perinatology, Women and Reproductive Health (CLAP/WR), Montevideo, Uruguay; 2 Department of Obstetrics and Gynecology, University of Campinas, Campinas, SP, Brazil; 3 Jundiaí School of Medicine - HU/FMJ, Jundiaí, SP Brazil; 4 Hospital Berta Calderon Roque, Managua, Nicaragua; 5 Hospital España, Chinandega, Nicaragua; 6 Hospital San Lorenzo de Los Mina, Santo Domingo , Dominican Republic; 7 Hospital Regional de Ocidente, Quetzaltenango, Guatemala; 8 Hospital San Felipe, Tegucigalpa, Honduras; 9 Hospital Roberto Suazo Cordova, La Paz, Honduras; 10 Hospital Leonardo Martinez Valenzuela, San Pedro Sula, Honduras; 11 Hospital Boliviano Japones, El Alto, Bolivia

**Keywords:** Severe maternal morbidity, Cesarean section, Robson classification

## Abstract

**Background:**

Latin America has the highest Cesarean Section Rates (CSR) in the world. Robson’s Ten Group Classification System (RTGCS) was developed to enable understanding the CSR in different groups of women, classified according to obstetric characteristics into one of ten groups. The size of each CS group may provide helpful data on quality of care in a determined region or setting. Data can potentially be used to compare the impact of conditions such as maternal morbidity on CSR. The objective of this study is to understand the impact of Severe Maternal Morbidity (SMM) on CSR in ten different groups of RTGCS.

**Methods:**

Secondary analysis of childbirth information from 2018 to 2021, including 8 health facilities from 5 Latin American and Caribbean countries (Bolivia, Guatemala, Honduras, Nicaragua, and the Dominican Republic), using a surveillance database (SIP-Perinatal Information System, in Spanish) implemented in different settings across Latin America. Women were classified into one of RTGCS. The frequency of each group and its respective CSR were described. Furthermore, the sample was divided into two groups, according to maternal outcomes: women without SMM and those who experienced SMM, considering Potentially Life-threatening Conditions, Maternal Near Miss and Maternal Death as the continuum of morbidity.

**Results:**

Available data were obtained from 92,688 deliveries using the Robson Classification. Overall CSR was around 38%. Group 5 was responsible for almost one-third of cesarean sections. SMM occurred in 6.7% of cases. Among these cases, the overall CSR was almost 70% in this group. Group 10 had a major role (preterm deliveries). Group 5 (previous Cesarean section) had a very high CSR within the group, regardless of the occurrence of maternal morbidity (over 80%).

**Conclusion:**

Cesarean section rate was higher in women experiencing SMM than in those without SMM in Latin America. SMM was associated with higher Cesarean section rates, especially in groups 1 and 3. Nevertheless, group 5 was the major contributor to the overall CSR.

## Background

Cesarean surgery is a potentially life-saving intervention. Nevertheless, C-section rates higher than 15% fail to improve maternal, fetal or perinatal outcomes, according to the World Health Organization (WHO) [1]. Robson’s Ten Group Classification System (RTGCS) has been developed to understand Cesarean section rates (CSR) in different groups of women, classified according to their obstetrics characteristics into one of the ten groups [2].

RTGCS considers the following six maternal characteristics to discriminate a woman admitted for childbirth into one (and only one) group: parity, previous cesarean section, gestational age, fetal presentation, onset of labor and number of fetuses. RTGCS is an easy-to-use tool since the variables considered are currently and routinely obtained in regular obstetric practice [3]. The size of each group and its CSR may provide helpful data on the quality of care in a determined region or setting. It can potentially be used to compare the impact of interventions or certain conditions (such as maternal morbidity) on CSR.

In Latin America, CSR is estimated to be 42.8%, the highest rate among all world regions [4]. Cesarean section was associated with maternal death (MD) [PR 1.99 (1.34–2.95)] and maternal near miss (MNM) [PR 3.40 (2.80–4.14)] [5] in the region. However, data on the impact of severe maternal morbidity (SMM) and maternal near miss (MNM) on cesarean section is scarce.

The Latin American Center of Perinatology (CLAP) is a branch of the Department of Family, Gender and Life Course of the Pan-American Health Organization, a WHO regional office. In the last 25 years, the CLAP has built and continuously improved the SIP (Perinatal Information System, in Spanish). SIP is a web-based database that gathers information on pregnancy outcomes from Latin American and Caribbean facilities. The SIP was created for surveillance of maternal health, rather than specifically for research purposes. However, its database has already been tested by our group to provide information on maternal morbidity [5]. The Latin American Center of Perinatology (CLAP) coordinates a network of sentinel centers in Latin American and Caribbean countries for the surveillance of maternal health related issues. All centers use SIP-PLUS as a common data collection system. The SIP forms cover information on demographic characteristics, obstetric information and childbirth data.

SIP database contains all variables necessary to classify a woman into the RTGCS, and more recently, in the RedCLAP context that introduced the concept of surveillance of maternal morbidity and also gathers routine information on SMM. Therefore, SIP database can provide valuable information about the impact of SMM and MNM on cesarean section rates in each of the ten groups of RTGCS.

This analysis was aimed at describing the impact of SMM on cesarean section rates, in ten different groups of the RTGCS.

## Methods

A secondary cross-sectional analysis of the SIP database was performed. It covered a time frame of 3 years (from 2018 to 2021) and included 8 health facilities from 5 Latin American and Caribbean countries (Bolivia, Guatemala, Honduras, Nicaragua, and the Dominican Republic). The study entered the RedCLAP [6] initiative, following a standardized protocol. Data was extracted from SIP servers and was analyzed after a detailed process of data management and consistency checking. Excluded from analysis were women whose data were missing for classification into the RTGCS or mode of delivery.

A woman was classified into one of the RTGCS, on the basis of the following variables: parity; previous cesarean section; onset of labor; gestational age at birth; fetal presentation and number of fetuses. Table 1 describes each group.

**Table 1:** Robson’s Classification and characteristics of women included per group 2,3

Group	Characteristics
1	Nulliparous, single cephalic, ≥ 37 weeks, spontaneous labor
2	Nulliparous, single cephalic, ≥ 37 weeks, induced or CS before labor
3	Multiparous (excluding previous CS), single cephalic, ≥ 37 weeks, spontaneous labor
4	Multiparous (excluding previous CS), single cephalic, ≥ 37 weeks, induced or CS before labor
5	Previous CS, single cephalic, ≥ 37 weeks
6	All nulliparous breeches
7	All multiparous breeches, including previous CS
8	All multiple pregnancies, including previous CS
9	All abnormal lies, including previous CS
10	All single cephalic, < 37 weeks, including previous CS

CS: cesarean section

The rate of each group and its respective CSR were described. Then, the sample was divided into two groups, according to maternal outcomes: women who experienced SMM and those without SMM.

SMM patient is defined as “a woman who nearly died but survived a complication that occurred during pregnancy, childbirth or within 42 days of termination of pregnancy.” The SMM group included all women who experienced potentially life-threatening conditions (PLTC) or severe maternal outcomes (SMO) comprising maternal near miss (MNM) or maternal death (MD). The definition of SMM, PLTC and MNM followed WHO criteria [7].

The WHO defined PLTC as the occurrence of hemorrhagic disorders (abruptio placentae, accreta/increta/percreta placenta, ectopic pregnancy, postpartum hemorrhage, ruptured uterus), hypertensive disorders (severe preeclampsia, eclampsia, severe hypertension, hypertensive encephalopathy, HELLP syndrome), other systemic disorders (endometritis, pulmonary edema, respiratory failure, seizures, sepsis, shock, thrombocytopenia, thyroid crisis), or severe management indicators (blood transfusion, central venous access, hysterectomy, intensive care unit admission, hospital stay longer than 7 days after delivery, non-anesthetic intubation, return to operating room) [7].

MNM criteria involves organ dysfunction or failure and were based on clinical criteria (acute cyanosis, loss of consciousness lasting more than 12 h or accompanied by the absence of pulse/heartbeat, gasping, stroke, oliguria nonresponsive to fluids or diuretics, uncontrollable fit/total paralysis, jaundice in the presence of preeclampsia, clotting failure), laboratory-based criteria (oxygen saturation > 90% for > 60 min, pH < 7.1, PaO2/FiO2 < 200, lactate > 5, creatinine > 3.5 mg/dL, acute thrombocytopenia, bilirubin > 6.0 mg/dL, loss of consciousness associated with the presence of glucose and ketoacids in urine) or management-based criteria (use of continuous vasoactive drugs, intubation and ventilation > 60 min unrelated to anesthesia, hysterectomy following infection or hemorrhage, dialysis for acute renal failure, transfusion of > 5 red cell units, cardiopulmonary resuscitation) [7].

The rate per group and cesarean section rates for both groups were estimated and compared, considering the continuum of morbidity (PLTC and SMO). Then CSR and group sizes were compared between the group of no morbidity with those with PLTC and SMO using χ^2^ test and estimation of Prevalence Ratio (PR) with their respective 95% Confidence Interval (CI). To compare cesarean section rates among women with no morbidity, PLTC, and SMO, we considered the no morbidity group as the reference group. The PR was obtained by dividing the cesarean section rate in each RTGCS group between women with no morbidity and those with PLTC, and the same for women with no morbidity and those with SMO. We performed the same to obtain PR regarding group size.

This study is a secondary analysis of the “Study on the incidence of severe maternal morbidity and mortality in maternities from the Red-CLAP from Latin America and the Caribbean”, approved by the Research Ethics Committee (REC) of the Pan American Health Organization (PAHO) in August 17, 2018 (PAHOERC, under number: PAHO-2018-04-0025).

## Results

**Fig. 1:**
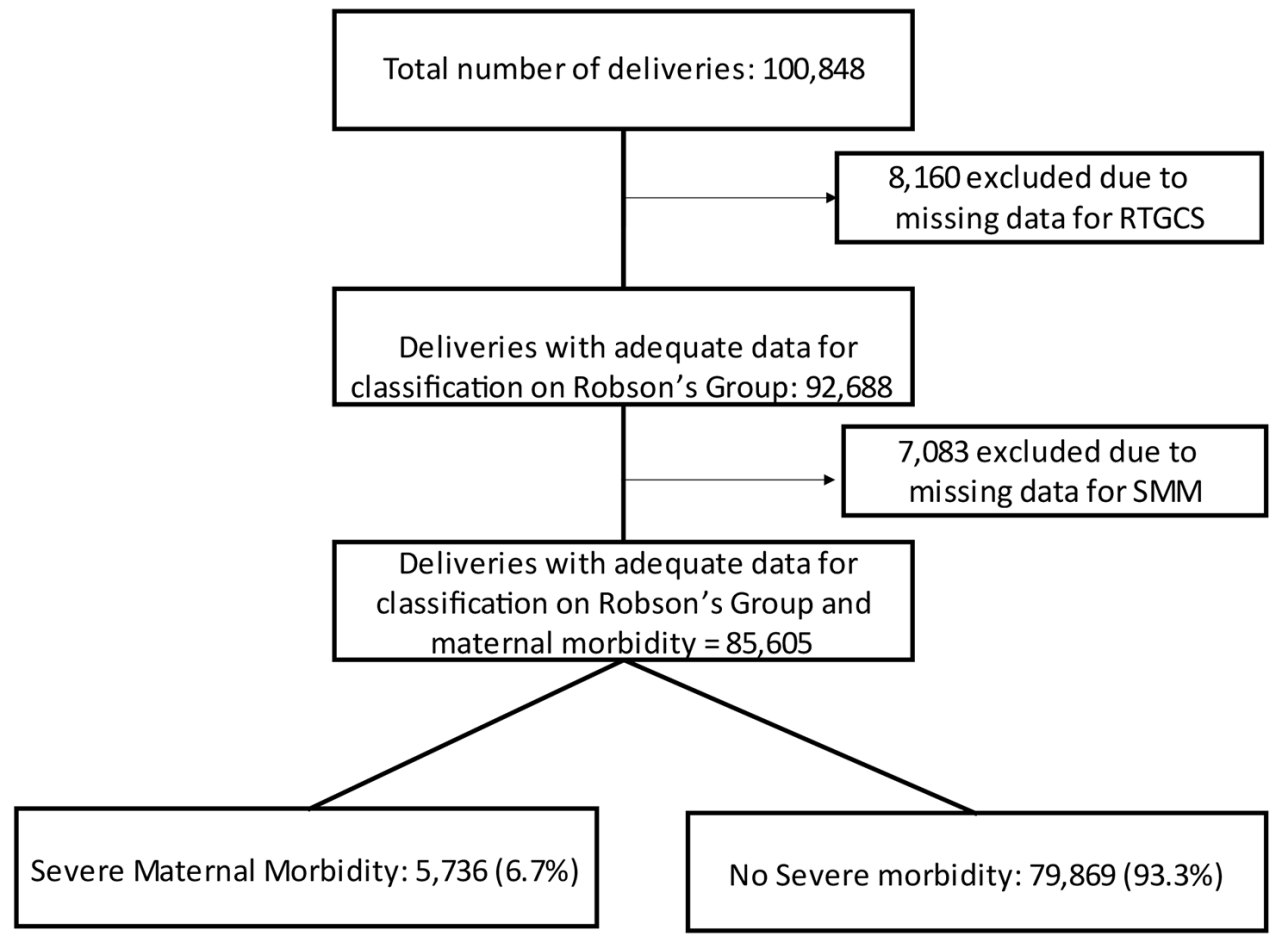
Flowchart of inclusion of cases in the study

Data on a total of 100,848 deliveries were retrieved from the SIP database. For initial analysis, 8,160 (8.1%) were excluded due to unavailable data that would allow classification into Robson’s groups. Of the total number of deliveries, 85,605 (84.9%) had reliable data. Therefore, it was possible to classify women into Robson’s groups and evaluate maternal morbidity. Furthermore, additional 7,083 (7.02%) of the deliveries considered were excluded from the study. Women were then divided into two groups: (1) SMM, with 5,736 (6.7%) deliveries, and (2) no severe morbidity, with 79,869 (93.3%) deliveries. Figure 1 shows the flowchart of included cases.

Table 2 shows data on 92,688 deliveries that had available data on the Robson Classification System. The overall CSR was 38.13%. Groups 1 to 4 accounted for 73.5% of all deliveries and 48.3% of all cesarean sections. On the other hand, group 5 contributed to 14.3% of all deliveries and 32.3% of all cesarean sections. The preterm birth rate was 8.0% and the CSR was 47.2% in those deliveries. The remaining 4 groups (6 to 9) accounted for 4.2% of all cases, with an expected high CSR (over 80%). The lowest cesarean section rate was observed in group 3 (8.9%), while group 5 (women with at least one previous cesarean section) had a CSR of 86.3%.

**Table 2:** Distribution of all women included in the study in the ten groups of Robson’s Classification, cesarean section rate in each group, and contribution of each group to overall cesarean section rate

Group	Number of CS in group	Number of women in group	Group size (%)	Group CS rate (%)	Absolute group contribution to overall CS rate	Relative contribution of group to overall CS rate
1	5.434	25.991	28.04%	20.91%	5.86%	15.38%
2	6.191	8.589	9.27%	72.08%	6.68%	17.52%
3	2.524	28.477	30.72%	8.86%	2.72%	7.14%
4	2.927	5.090	5.49%	57.50%	3.16%	8.28%
5	11.422	13.238	14.28%	86.28%	12.32%	32.32%
6	784	854	0.92%	91.80%	0.85%	2.22%
7	871	981	1.06%	88.79%	0.94%	2.46%
8	1.246	1.503	1.62%	82.90%	1.34%	3.53%
9	437	544	0.59%	80.33%	0.47%	1.24%
10	3.502	7.421	8.01%	47.19%	3.78%	9.91%
Total	35.338%	92.688%	100%	38.13%		100%

Table 3 demonstrates data on women with SMM. The overall CSR was 67.9% in women with SMM. Among those women, group 10 (women with preterm delivery) was the largest group (25.8%), with a cesarean section rate of 79.9%, while groups 1 to 4 account for 53.2% of all deliveries. Only two groups had cesarean section rates below 50% (groups 1 and 3). Groups 10 and 5 were the highest contributors to cesarean section rates.

**Table 3:** Distribution of women with SMM (PLTC + MNM) + MD in Robson’s Ten Group Classification (RTGCS), cesarean section rate per group, and contribution of each group to overall cesarean section rate

Group	Number of CS in group	Number of women in group	Group size (%)	Group CS rate (%)	Absolute group contribution to overall CS rate	Relative contribution of group to overall CS rate		
1	539	1.146	19.98%	47.03%	9.40%		13.83%	
2	560	651	11.35%	86.02%	9.76%		14.37%	
3	266	930	16.21%	28.60%	4.64%		6.83%	
4	253	323	5.63%	78.33%	4.41%		6.49%	
5	661	740	12.90%	89.32%	11.52%		16.97%	
6	62	68	1.19%	91.18%	1.08%		1.59%	
7	72	81	1.41%	88.89%	1.26%		1.85%	
8	255	271	4.72%	94.10%	4.45%		6.55%	
9	43	44	0.77%	97.73%	0.75%		1.10%	
10	1.185	1.482	25.84%	79.96%	20.66%		30.42%	
Total	3.896	5.736	100%	67.92%			100%	

SMM: Severe maternal morbidity; PLTC: potentially life-threatening conditions; MNM: maternal near miss; MD: maternal death.

Among women without SMM, group 5 (women with at least one previous cesarean section) contributed with the highest cesarean section rates (34.3%), and the overall cesarean section rate was 36.5%. On the other hand, women who experienced spontaneous labor and had a previous vaginal delivery (group 3), accounted for the largest group (31.5% of all cases) with a cesarean section rate of 8.1% (Table 4).

**Table 4:** Distribution of women without SMM in Robson’s Ten Group Classification (RTGCS), cesarean section rate per group, and contribution of each group to overall cesarean section rate

Grupo	Number of CS in group	Number of women in group	Group size (%)	Group CS rate (%)	Absolute group contribution to overall CS rate	Relative contribution of group to overall CS rate
1	4.422	22.659	28.37%	19.52%	5.54%	15.15%
2	5.451	7.680	9.62%	70.98%	6.82%	18.68%
3	2.051	25.159	31.50%	8.15%	2.57%	7.03%
4	2.569	4.612	5.77%	55.70%	3.22%	8.80%
5	10.008	11.439	14.32%	87.49%	12.53%	34.29%
6	670	728	0.91%	92.03%	0.84%	2.30%
7	750	831	1.04%	90.25%	0.94%	2.57%
8	906	1.112	1.39%	81.47%	1.13%	3.10%
9	369	445	0.56%	82.92%	0.46%	1.26%
10	1.990	5.204	6.52%	38.24%	2.49%	6.82%
Total	29.186	79.869	100%	36.54%		100%

SMM: Severe maternal morbidity.

We also compared the prevalence ratio of cesarean section between women with and without SMM. Those data are shown in Table 5. SMM increased the prevalence of cesarean section in groups 1 to 4 and groups 8 to 10. The likelihood of having a cesarean section increased 3.5-, 2.4- and 2.1-fold in groups 3, 1 and 10, respectively. Nevertheless, in women with at least one previous cesarean section (group 5), the same effect was not observed.

**Table 5:** Comparison of cesarean section in each RTGCS between women with no severe morbidity and those with severe maternal morbidity (SMM)

Group	No morbidity	PLTC	SMO	p-value/PR^1^	p-value/PR^2^
1	4422 (19.5)	516 (48.0)	23 (31.9)	**< 0.001 / 2.46 (1.82–3.33)**	0.059 / 1.64 (0.97–2.75)
2	5451 (71.0)	522 (86.1)	38 (84.4)	0.066 / 1.21 (0.98–1.50)	0.153 / 1.19 (0.92–1.54)
3	2051 (8.2)	244 (28.6)	22 (28.2)	**< 0.001 / 3.51 (2.17–5.68)**	**0.020 / 3.46 (1.29–9.25)**
4	2569 (55.7)	224 (78.3)	29 (78.4)	0.102 / 1.41 (0.92–2.16)	0.138 / 1.41 (0.87–2.28)
5	10,008 (87.5)	603 (90.0)	58 (82.9)	0.472 / 1.03 (0.94–1.12)	0.406 / 0.95 (0.82–1.10)
6	670 (92.0)	58 (93.5)	4 (66.7)	0.467 / 1.02 (0.97–1.07)	0.487 (0.72 (0.25–2.10)
7	750 (90.3)	59 (89.4)	13 (86.7)	0.796 / 0.99 (0.91–1.08)	0.695 / 0.96 (0.75–1.22)
8	906 (81.5)	229 (93.9)	26 (96.3)	0.120 / 1.15 (0.95–1.39)	**0.025 / 1.18 (1.03–1.36)**
9	369 (82.9)	35 (100)	8 (88.9)	**0.007 / 1.21 (1.07–1.35)**	0.515 / 1.07 (0.84–1.37)
10	1990 (38.2)	1033 (80.4)	152 (77.2)	**< 0.001 / 2.10 (1.62–2.73)**	**< 0.002 / 2.02 (1.51–2.69)**
Overall CSR	29,186 (36.5)	3523 (68.0)	373 (67.1)	**< 0.001 / 1.86 (1.82–1.90)**	**< 0.001 / 1.83 (1.73–1.95)**

1 – p-value refers to the comparison between no morbidity and PLTC; 2 – p-value refers to the comparison between no morbidity and SMO.

Regarding the overall size of each group, and in the comparison between cases with and without SMM, the higher rate of group 10 (preterm birth) was the most significant finding. Group 10 was responsible for 6.5% of cases without SMM, as well as 24.8% and 35.4% in PLTC and SMO cases, respectively (Table 6).

**Table 6:** Comparison of group size in each of RTGCS between women with no morbidity and those with PLTC or SMO

Group	No morbidity	Group size (%)	PLTC	Group size (%)	SMO	Group size (%)	p-value/PR^1^	p-value/PR^2^
1	22.659	28.4	1074	20,7	72	12,9	**< 0.01 / 0.73 (0.69–0.77)**	**< 0.01 / 0.45 (0.37–0.57)**
2	7.680	9.6	606	11,7	45	8,1	**< 0.01 / 1.22 (1.12–1.31)**	0.22 / 0.84 (0.63–1.11)
3	25.159	31.5	852	16,4	78	14,0	**< 0.01 / 0.52 (0.49–0.55)**	**< 0.01 / 0.44 (0.36–0.55)**
4	4.612	5.8	286	5,5	37	6,7	0.44 / 0.96 (0.85–1.07)	0.37 / 1.15 (0.84–1.57)
5	11.439	14.3	670	12,9	70	12,6	**< 0.01 / 0.90 (0.83–0.97)**	0.24 / 0.88 (0.70–1.09)
6	728	0.9	62	1,2	6	1,1	**0.04 / 1.31 (1.01–1.70)**	0.68 / 1.18 (0.52–2.63)
7	831	1.0	66	1,3	15	2.7	0.11 / 1.22 (0.95–1.57)	**< 0.01 / 2.59 (1.56–4.29)**
8	1.112	1.4	244	4.7	27	4,9	**< 0.01 / 3.38 (2.95–3.87)**	**< 0.01 / 3.49 (2.40–5.06)**
9	445	0.6	35	0,7	9	1,6	0.26 / 1.21 (0.86–1.71)	**< 0.01 / 2.90 (1.50–5.59)**
10	5.204	6.5	1285	24,8	197	35,4	**< 0.01 / 3.33 (3.15–3.52)**	**< 0.01 / 5.44 (4.85–6.10)**

1 – p-value refers to the comparison between no morbidity and PLTC; 2 – p-value refers to the comparison between no morbidity and SMO.

## Discussion

This study demonstrates data on the use of RTGCS to evaluate CSR, comparing cases with and without SMM, using a surveillance database implemented in different settings across Latin America. Overall, CSR was around 38%, with group 5 responsible for almost one-third of cesarean sections. There were 6.7% of SMM cases. Of these cases, the overall CSR was almost 70% and group 10 (preterm deliveries) had a major role (preterm deliveries). It makes sense to perform a cesarean section when complications or morbidities arise. In most cases, the mode of delivery is optional and childbirth may occur during the preterm period. However, more emphasis should be given to labor induction (at least in late preterm labor). Group 5 had a very high CSR within the group, regardless of the presence of maternal morbidity (over 80%).

CSR was assessed in each of the 10 groups by the Robson classification. In each group it is important to evaluate the contribution to overall CSR, since larger groups, even those with lower CS rates, can potentially have a high overall impact on increased CSR. The Robson classification is a tool that compares the quality of obstetric care. Recently, it has also enabled auditing of important obstetric outcomes, other than C-Sections in all Robson groups [8, 9] including severe maternal morbidity [10].

Studies from Brazil obtained results similar to findings in our study, where CSR is 73.2% in SMM cases. Group 5 had a relevant impact and the CSR within the group achieved 84.6% among severe morbidity cases [10]. It is important to note that in the general population, group 5 is also the largest group in Brazil with the highest CSR (a group size of 21.7%, CSR of 84.9%, contributing to a CSR of 18.4%) [11]. Although a previous cesarean section is not determinant of a new cesarean section, it is very unlikely that a woman with a previous cesarean section will undergo vaginal delivery in a new pregnancy [12], unless she personally requests the mode of delivery and she finds an obstetric team that supports her decision. If she experiences SMM, the chances of undergoing vaginal delivery are limited, as suggested by our data. Avoiding the first C-section seems to be the initial key factor for reducing CSR in Latin America, even in cases of SMM where a cesarean section is not mandatory.

Our overall CSR was similar to those shown in a study performed in Latin America from 2004 to 2005 [13]. That study included 97,095 deliveries that occurred in 120 facilities from 8 Latin American countries, and found a CSR of 35.4%, while our rate was 38.1%. The CSR and size of each group were similar in both studies. Group 5 was the main contributor to overall CSR. RTGCS is a useful tool to compare different time periods, and it seems that no significant increasing trend in CSR has occurred in Latin America between both time periods (13 years apart).

Other studies that evaluated CSR considering RTGCS in Latin America have shown that CSR is high in countries, when analyzed separately. A Uruguayan study, also using routine information gathered with SIP, found an overall CSR of 43.1%. In that study, Group 5 had the largest size and was the major contributor to overall CSR (30.6%) [14]. Another Mexican study found an overall CSR ranging from 53.5 to 48.7% [15]. Only a Chilean facility-based study found lower rates (27%), despite drawing attention to the increase in C-sections in groups 1, 3 and 4, suggesting that in the following years the facility will probably face an increase in group 5 [16].

More important than achieving a specific CSR is providing the necessary cesarean sections according to maternal or fetal needs, especially in low- and middle-income countries. The reduction of CSR may be considered alongside other outcomes, such as neonatal near miss and death [17]. Our study showed that considering maternal outcomes such as SMM can be evaluated together with CSR – and it is important to provide adequate and timely efficient care for women with SMM.

Cesarean section is a useful intervention to reduce neonatal and perinatal death in pregnancies with gestational age lower than 32 weeks and breech fetuses; however, the same protective effect was not observed among cephalic fetuses of similar gestational age [18]. Among women with SMM, 25.8% were included in group 10, which comprises preterm deliveries with cephalic fetuses, with a CSR of almost 80% - and the group was responsible for the highest relative contribution to overall CSR (30.4%). Among women without SMM, the CSR was also high (47.2%). It is important to disseminate the evidence regarding route of delivery, especially among women with SMM, avoiding exposing them to unnecessary surgical interventions.

Our SMM rate (6.7%) was higher than the rate observed in a systematic review that contained 8 studies (2.45%) [19]. However, it was close to the only Latin American study included in the metanalysis-a study from the Northeast of Brazil, with a SMM rate of 6.8% [20]. The same systematic review observed that women with a previous cesarean section had a 1.6-fold chance of experiencing SMM, and a 2.7-fold chance of experiencing MNM. In our study, we found that women in groups 1 and 3 (no previous cesarean) were more likely to undergo cesarean section in the current pregnancy. The combination of two factors (history of previous cesarean section and SMM in a previous pregnancy) may probably have an impact on the next pregnancy, suggesting that special care is required in these women.

The use of the RTGCS has increased and its understanding expanded in the last years; a Swedish study showed an overall CSR of 17.4%, and that study analyzed other outcomes such as operative vaginal delivery, obstetric anal sphincter injury and postpartum haemorrhage [8]. A Brazilian study showed that, among women with SMM, 73.2% underwent CS – and group 10 was the most prevalent (33.9%) and responsible for majority of cesarean deliveries (38.2%), with a group CSR of 82.6% [10]. Another Brazilian study compared CSR between women with and without preeclampsia, showing that women with preeclampsia were 2.3 times more likely to undergo cesarean Sect [9]. Those studies suggest that RTGCS can be a useful tool to understand not only CSR and obstetric populations but also other important conditions on obstetric daily practice.

Our study has some limitations. The tool used to generate the database was not primarily designed to be used for scientific purposes but mainly for clinical practice and surveillance and therefore some information is not available. On the other hand, this database contains data from almost 100,000 women who gave birth in 5 different Latin American countries and the experience could be expanded to other countries and facilities using the same SIP platform, what would include millions of deliveries, then providing more robust evidence.

## Conclusions

In Latin America, cesarean section rates were higher in women experiencing SMM than in those without SMM. SMM was associated with the performance of cesarean section, particularly in groups 1 and 3. Group 5, however, was the major contributor to overall CSR.

## Data Availability

The property of data used in this manuscript is of each participating country, coordinated by the PAHO-CLAP in Montevideo, Uruguay. The data can be available from there upon a reasonable request to the director of CLAP-PAHO (serruyas@paho.org).

## List of abbreviations

CLAP: Latin American Center of Perinatology
CS: Cesarean Section
CSR: Cesarean Section Rates
MD: Maternal Death
MNM: Maternal Near Miss
PLTC: Potentially Life-Threatening Condition
PR: Prevalence Ratio
REC: Research Ethics Committee
RTGCS: Robson’s Ten Group Classification System
SIP: Perinatal Information System
SMM: Severe Maternal Morbidity
SMO: Severe Maternal Outcome
WHO: World Health Organization
